# Chlorogenic Acid Alleviates Hepatic Ischemia–Reperfusion Injury by Inhibiting Oxidative Stress, Inflammation, and Mitochondria-Mediated Apoptosis *In Vivo* and *In Vitro*

**DOI:** 10.1007/s10753-023-01792-8

**Published:** 2023-03-01

**Authors:** Kai Li, Zanjie Feng, Liusong Wang, Xuan Ma, Lei Wang, Kangwei Liu, Xin Geng, Cijun Peng

**Affiliations:** 1 Department of Hepatobiliary and Pancreatic Surgery, Affiliated Hospital of Zunyi Medical University, Zunyi, Guizhou, China; 2 Department of Biochemistry and Molecular Biology, Zunyi Medical University, Zunyi, Guizhou, China; 3 Department of Hepatobiliary and Pancreatic Surgery, The People’s Hospital of Jianyang City, Jianyang, China

**Keywords:** Chlorogenic acid, Hepatic ischemia–reperfusion injury, Inflammation, Oxidative stress, Mitochondria

## Abstract

Hepatic ischemia–reperfusion injury (HIRI) is the main reason for organ failure following liver surgery; however, its underlying causes are complex, and include oxidative stress, sterile inflammatory, and mitochondrial damage. Unfortunately, treatments for HIRI are based on supportive therapy, and no specific drugs or methods are currently available. Chlorogenic acid (CGA) is a dietary polyphenol with a wide range of pharmacological effects and it has a protective effect on HIRI; however, its specific mechanism remains unclear. In this study, we investigated that CGA pretreatment exerts protective effects against HIRI and the potential underlying mechanisms. We found that CGA pretreatment reduced ALT, AST, MDA, TNF-α, and IL-1β levels following HIRI, improved SOD and GSH levels, and alleviated pathological liver tissue damage, with the highest CGA dose (100 mg/kg.d) exerted the strongest effect. In addition, we showed that CGA pretreatment significantly decreased the levels of reactive oxygen species following HIRI, inhibited HMGB1 release by decreasing IRF-1 expression, inhibited the expression of HMGB1, TLR-4, MyD88, P-IκB-α, NF-κB P65, and P-P65, and promoted IκB-α degradation. Thus, CGA appears to inhibit oxidative stress and inflammatory responses during HIRI. Furthermore, we found that CGA pretreatment reduced hepatocyte apoptosis following HIRI, alleviated mitochondrial damage, promoted BCL-2 expression, inhibited Bax upregulation, and inhibited cytochrome C release to prevent caspase activation, thereby reducing the expression of the caspase-independent pathway components, ENDOG and AIF. Together, our findings suggest that CGA can protect against HIRI by inhibiting oxidative stress, the HMGB1/TLR-4/NF-κB signaling pathway–mediated inflammatory responses, and mitochondria-mediated apoptosis. Thus, CGA appears to be a promising therapeutic approach for treating HIRI.

**Supplementary Information:**

The online version contains supplementary material available at 10.1007/s10753-023-01792-8.

## INTRODUCTION

Hepatic ischemia–reperfusion injury (HIRI) occurs during liver transplantation, liver resection, and other liver surgeries, and is an important cause of early transplantation failure, tissue injury, organ rejection, and even liver failure [1]. The complex pathological process of HIRI is not well studied, and include oxidative stress, aseptic inflammatory, and apoptosis [2]. However, no pharmacological approaches have been approved for the prevention or treatment of HIRI [3], so it remains a significant challenge for clinicians.

Although multiple studies have investigated the potential mechanisms of HIRI, they currently remain unclear. Reactive oxygen species (ROS) is one of the most prominent factors of HIRI causing liver injury and is mainly produced by Kupffer cells and mitochondria during ischemia–reperfusion (I/R) [4]. Glycogen depletion and ATP deficiency during ischemia does not pose a risk to the liver; however, the acute and massive ROS release, as well as inflammation that occur during reperfusion can cause extensive hepatocellular injury and necrosis [4, 5]. Studies have shown that the scavenging of excessive ROS will be beneficial to mitigate the sterile inflammatory response and apoptosis [6, 7]. Therefore, excessive ROS production is one of the earliest and most important factors that contribute toward liver damage during HIRI.

Numerous studies have shown that ROS is associated with the early production of endogenous damage-associated molecular patterns (DAMPs); high mobility group box 1 (HMGB1), one of the most studied DAMPs, mediates the complex aseptic inflammatory response [8]. HMGB1 normally binds to nuclear DNA in its deacetylated form; however, during I/R, ROS promote the expression of IRF-1, an early response transcription factor that can enhance histone acetyl transferase activity and promote the acetylation of HMGB1. Acetylated (Ac)-HMGB1 then dissociates from nuclear DNA and enters the cytoplasm, where intracellular Ca^2+^ can promote the loading of HMGB1 into lysosomes for extracellular secretion [9–11]. Ac-HMGB1 mainly recognizes and binds to the Toll-like receptor 4 (TLR-4) receptor which then activates nuclear factor-κB (NF-κB) to mediate sterile inflammatory responses [12]. In addition, accumulating evidence has suggested that both inflammatory responses and I/R injury are attenuated in the absence of TLR-4 [13]. Therefore, HMGB1/TLR-4 pathway blockade could be a therapeutic target for treating HIRI.

Recent evidence has indicated that apoptosis is the main mode of I/R-induced hepatocyte death and that mitochondrial function exerts a key role in HIRI development [14, 15]. Animal studies have also shown that infusion with active mitochondria before reperfusion can significantly reduce HIRI [16] and that inhibiting apoptosis can reduce the degree of IRI in the liver and heart [17]. ROS-induced oxidative stress plays a key role in cell apoptosis and promotes mitochondrial damage and apoptosis signaling pathways during HIRI, especially in the early stages [18]. Excessive ROS accumulation in the liver after I/R can lead to mitochondrial crest loss, decreased mitochondrial membrane potential, and disrupted the balance of pro-apoptotic (Bad, Bax, and Bid) and anti-apoptotic (BCL-2 and BCL-XL), enhanced mitochondrial permeability transition pore opening, causing cytochrome C (Cyt-c) spillover, and initiation of endogenous apoptosis [19, 20]. Several drugs have recently been reported to reduce ROS induction by HIRI, thereby reducing hepatocyte apoptosis [21–23]. Consequently, ROS links and integrates the complex inflammatory response and mitochondrial damage-mediated apoptosis, and provides a new avenue for its prevention and treatment.

Traditional Chinese medicines have been used for many years and have a wide range of application, low toxicity, and few side effects. However, there has been no breakthrough in the protective effect of drug pretreatment for HIRI, thus it is important to develop herbal medicines or monomers with protective effects against HIRI. Chlorogenic acid (CGA), also known as 5-O-caffeoylquinic acid (Fig. S1), is widely found in fruits, vegetables, and spices, and is one of the most abundant and powerful polyphenol compounds in the human diet [24]. It has many important effects such as antioxidant, antibacterial, hepatoprotective, cardioprotective, and anti-inflammatory properties [25]. CGA ameliorates alcohol-induced liver injury by scavenging ROS and inhibits CCl_4_-induced liver fibrosis in rats, potentially by being related to TLR-4/MyD88/NF-κB signaling pathway inhibition [26, 27]. Furthermore, CGA has demonstrated protective effects against oxidative stress, inflammation, and apoptosis in methotrexate-induced rat liver injury [28]. Since CGA can protect against liver injury via mechanisms related to ROS removal, the inhibition of inflammation, and apoptosis, we aimed to investigate the protective effects of CGA against HIRI as well as its potential underlying mechanisms to identify potential new targets for the prevention and treatment of HIRI.

## MATERIALS AND METHODS

### Animals

Forty male Sprague-Dawley rats (160–200 g; SPF grade) were purchased (Tianqin, Changsha, China) and housed in the animal center (Zunyi Medical University, Zunyi, China) under an ambient temperature of 21—27 °C at 50% humidity. All rat experiments were approved by the Laboratory Animal Welfare & Ethics Committee of Zunyi Medical University (approval no: KLLY(A)-2020-029).

#### Animal Grouping and Pretreatment

CGA was obtained from Huamike Biological Co., Ltd (Beijing, China; purity > 98%). The rats were randomly assigned to five groups: the sham-operated group (Sham, *n* = 8), I/R + low-dose CGA group (I/R + CGA-L, *n* = 8), I/R + medium-dose CGA group (I/R + CGA-M,* n* = 8), I/R + high-dose CGA group (I/R + CGA-H,* n* = 8), and the ischemia–reperfusion group (I/R,* n* = 8). The I/R + CGA-L, I/R + CGA-M, and I/R + CGA-H groups were administered 20 mL/kg.d CGA by gavage at concentrations of 25, 50, and 100 mg/kg.d for 10 days, respectively, as reported by previous studies [29, 30]. The Sham and I/R groups were administered 20 mL/kg.d normal saline by gavage for 10 days.

#### Rat HIRI Model

The rat HIRI model was established as described previously [31]. The rats were anesthetized by intraperitoneal injection of 1% pentobarbital (50 mg/kg, obtained from the Affiliated Hospital of Zunyi Medical University), and all groups, except for the Sham group, were dissected at the hepatoduodenal ligament, then, non-invasive vascular clips were used to block the vessels and bile ducts leading to the middle and left lobes of the liver, thus causing 70% of the liver ischemia. Thereafter, the ischemic liver lobes changed color from bright red (Figure S2A) to grayish white (Fig. S2B, C). The vascular clips were released after 1 h and the liver was reperfused for 4 h (I/R treatment times were based on the method of Gao W et al. [32]), and the color gradually changed from gray to bright red (Fig. S2D), indicating a successful I/R model. In the Sham group, the hepatoduodenal ligament was dissected but hepatic blood flow was not blocked.

### Cell Culture and Transfection

Normal human hepatocytes (HL7702, L02) were obtained from the Cell Bank of Chinese Academy of Sciences (Shanghai, China), thawed, and cultured in RPMI-1640 medium containing 100 U/mL penicillin, 100 μg/mL streptomycin, and 10% fetal bovine serum at 37 °C in an incubator with 5% CO_2_. Some of the cells were inoculated in six-well plates (1 × 10^5^ cells/well) and cultured in complete RPMI-1640 medium. Upon reaching 50% confluence, L02 cells were transfected for 48 h with TLR-4 overexpression plasmids (NMID: NM_138554.1; HANBIO, Shanghai, China; MOI = 20) and empty plasmids using lentivirus; the transfection efficiency was increased using polybrene (6 μg/mL).

### Cell Proliferation Assay

The effects of CGA on L02 cells cytotoxicity and proliferation were detected using the Cell Counting Kit-8 assay kit (CCK-8; Dojindo, Beijing, China), according to the manufacturer’s instructions. The L02 cells were cultured overnight in 96-well plates (5 × 10^3^ cells/well). The following day, the cells were washed and replaced with medium containing different CGA concentrations and incubation was continued for 48 h. Thereafter, 10 μL of CCK8 reagent was added to each well and incubated at 37 °C for 2 h. Lastly, the absorbance at 450 nm was detected by an absorbance reader (Molecular Devices, CA, USA).

### Cell Grouping and Pretreatment

The cells were divided into 5 groups upon successful transfection of the TLR-4 overexpression plasmid: a normal L02 cell group (NC), a TLR-4 overexpression + CGA group (OE+CGA), a TLR-4 overexpression group (OE), a hypoxia/reoxygenation + CGA group (H/R+CGA), and a hypoxia/reoxygenation (H/R) group. The NC, OE and H/R groups were given complete medium, while the OE+CGA and H/R+CGA groups were given a mixture of CGA and complete medium, and they were incubated for 48 h at 37 °C with 5% CO_2_. On the third day, the H/R+CGA and H/R groups underwent hypoxia preadaptation for 6 h in a three-gas hypoxia incubator (Thermo, MA, USA) with N_2_ (94%), O_2_ (1%), and CO_2_ (5%) in sugar-free Dulbecco’s modified Eagle medium. Then the cells were replaced with complete RPMI-1640 medium and cultured in a 5% CO_2_ incubator at 37 ℃ for a further 12 h. The remaining 3 groups were simultaneously replaced with sugar-free Dulbecco’s modified Eagle medium and cultured in a 37 °C incubator with 5% CO_2_ for 6 h. Thereafter, they were replaced with complete medium, and incubation was continued for 12 h. Hypoxia (6 h) and reoxygenation (12 h) treatment times were based on the method of Huang et al. [2].

### Sample Collection

After liver reperfusion, the inferior vena cava of each rat was dissected and exposed and 4–6 ml blood was extracted. The serum was collected after blood coagulation, centrifuged for 10 min at 3000 rpm, and stored in a refrigerator at −80 °C for liver function analysis. Left liver lobe samples were taken uniformly from all rats and each sample was divided into several parts. The first part (0.5 cm × 0.5 cm) was stored in 10% formalin and sent to the Department of Pathology, Affiliated Hospital of Zunyi Medical University, where it was embedded in paraffin and cut into 5 μm-thick sections for TUNEL and hematoxylin-eosin (H&E) staining. The second part (0.5 cm × 0.5 cm) was cut into 10 μm-thick frozen sections for dihydroethidium (DHE) staining. The third part (0.1 g) was added to 1 mL of 0.9% saline, pulverized three times using a Xinyi-96 high-flux tissue grinder (Ningbo, China; 1500 rpm, 20 s). The ground liver tissue was allowed to stand 30 min and centrifuged for 10 min at 13,300 rpm, after which the supernatant was collected to obtain a 10% liver homogenate. The remaining part was stored at −80°C until analyzed.

### Determination of Liver Function and Detection of Oxidative Stress and Inflammatory Markers

The serum levels of alanine aminotransferase (ALT) and aspartate aminotransferase (AST) were analyzed by an AU5800 biochemical analyzer (Beckman Coulter, CA, USA). Superoxide dismutase (SOD), malondialdehyde (MDA) and glutathione (GSH) levels in liver homogenates, which are both oxidative stress markers, were measured using SOD, MDA and GSH kits (Njjcbio, Nanjing, China) according to the manufacturer’s instructions. The liver homogenate levels of the inflammatory markers HMGB1, interleukin-1β (IL-1β), and tumor necrosis factor alpha (TNF-α) were detected by enzyme-linked immunosorbent assay kits (LIANKE, Hangzhou, China) according to the manufacturer’s instructions.

### Histopathological Analysis

To evaluate liver tissue damage, paraffin sections were made and H&E staining was performed. The stained pathology sections were dried and examined by two experienced pathologists using a light microscope (Leica, Germany). According to Suzuki’s [33] criteria, histopathological scores (0–4) were given for each field: 0 = Normal hepatocytes; 1 = individual cellular edema, vacuolization, or single cellular necrosis; 2 = little cellular edema, vacuolization, or necrosis ≤ 30% ; 3 = moderate cellular edema, vacuolization, or necrosis ≤ 60%; and 4 = severe edema, vacuolization and necrosis > 60%.

#### Real-Time Quantitative PCR (RT-qPCR) Assays

Total RNA in liver specimens or cells were extracted using RNAiso Plus (TAKARA, Tokyo, Japan), quantified, and reverse-transcribed into cDNA. Target mRNA was amplified using standard two-step PCR and a reaction volume of 15 µL (including 7.5 μl TB Green Premix Ex Taq II, 1 μl specific forward primer, 1 μl specific reverse primer, 3 μl cDNA, and 2.5 μl sterile water). Thereafter, the 2^−ΔΔCq^ method was used to measure the gene relative expression levels and β-actin was used for normalization. Primer sequences for the target genes are shown in Table S1.

### Western Blotting Assays

The total protein was extracted from the collected cells or liver tissue samples using a lysis solution (RIPA buffer; Solarbio, Beijing, China), including protease inhibitors and phosphatase inhibitors, and then quantified using a BCA protein assay kit (Solarbio), according to the manufacturer’s instructions. Total proteins were separated by sodium dodecyl sulfate-polyacrylamide gels (8–15%) and transferred to polyvinylidene fluoride membranes. Non-specific binding was blocked using 5% non-fat milk (phosphorylated protein using 5% fetal bovine serum) for at least 1 h under room temperature. Next, the membranes and diluted primary antibodies were incubated overnight at 4 °C. The following day, the membranes were incubated with secondary antibodies (Proteintech, Wuhan, China; 1:2000) for at least 1 h at room temperature. The membranes were exposed to super sensitive ECL luminescence reagent (Meilunbio, Dalian, China) with an imaging system (Bio-Rad, CA, USA). The protein bands were analyzed, and their grayscale values were counted using Image Lab software (v.5.2; Bio-Rad). Details of the primary antibodies, including their source and working dilutions, are presented in Table S2.

### Immunoprecipitation (IP) Assays

The total protein was extracted by the same method as mentioned in the “Western Blotting Assays” section. Then, 1mg of the protein sample was mixed with 1 µg goat normal IgG and 20 µL of Protein A+G Agarose (Beyotime, Shanghai, China) and shaken slowly for 1.5 h at 4 °C. Thereafter, the supernatant was collected after centrifugation for 5 min at 2500 rpm to remove nonspecific binding; simultaneously, the goat normal IgG was used as a negative control. 1.5 µg of HMGB1 primary antibodies were added to each supernatant at 4 °C and shaken slowly overnight. The next day, another 40 µL Protein A+G Agarose was added, incubated for 2 h at 4 °C and centrifuged instantaneously at 13,300 rpm. After five washes with PBS, the supernatant was removed, and the sample was analyzed using western blotting.

### Fluorescence Analysis

ROS in the frozen liver sections was labeled with DHE (Beyotime), which produces red fluorescence. Apoptotic hepatocytes were detected using a mitochondrial membrane potential and apoptosis detection kit (Beyotime), according to the manufacturer’s instructions. Live cells with intact mitochondrial membrane potential were labeled with red fluorescence using MitoTracker Red CMXRos and apoptotic cells were labeled with green fluorescence using Annexin V- Fluorescein isothiocyanate (FITC). Nuclei were stained with blue fluorescence using 4,6-diamidino-2-phenylindole (DAPI) or Hoechst 33342. All steps were performed according to the instructions provided by the supplier. Next, the stained sections or cells were photographed under a fluorescent microscope (Olympus, Tokyo, Japan) and analyzed using Image-Pro Plus software (v.6.0; Bio-Rad).

### TUNEL Assays

A one-step TUNEL assay kit with FITC (Beyotime) was used to detect apoptosis in liver tissue. The slides were dewaxed and washed three times with PBS, incubated with proteinase K at 37 ℃ for 25 min, and then incubated with TUNEL reaction solution for 1 h in the dark. Next, 1 drop of anti-fluorescence quencher containing DAPI was added, and after 10 min the coverslip was covered and observed under a fluorescence microscope (Olympus). The fluorescence pictures were analyzed using Image-Pro Plus software (v.6.0; Bio-Rad).

### Statistical Analysis

All results were shown as means ± standard error (SEM) and were analyzed using SPSS (v.22) or GraphPad Prism software (v.8). One-way ANOVA was used for the comparison of data between groups conforming to normal distribution. Statistical significance was defined as *p* < 0.05. Since the histopathological grading data were not normally distributed, nonparametric comparisons were performed using the Kruskal–Wallis test. The level of significance for two-way comparisons between groups was defined as *p* < 0.005.

## RESULTS

### CGA improved liver function, oxidative stress, inflammation levels, and liver histopathology injury in rats

**Fig. 1:**
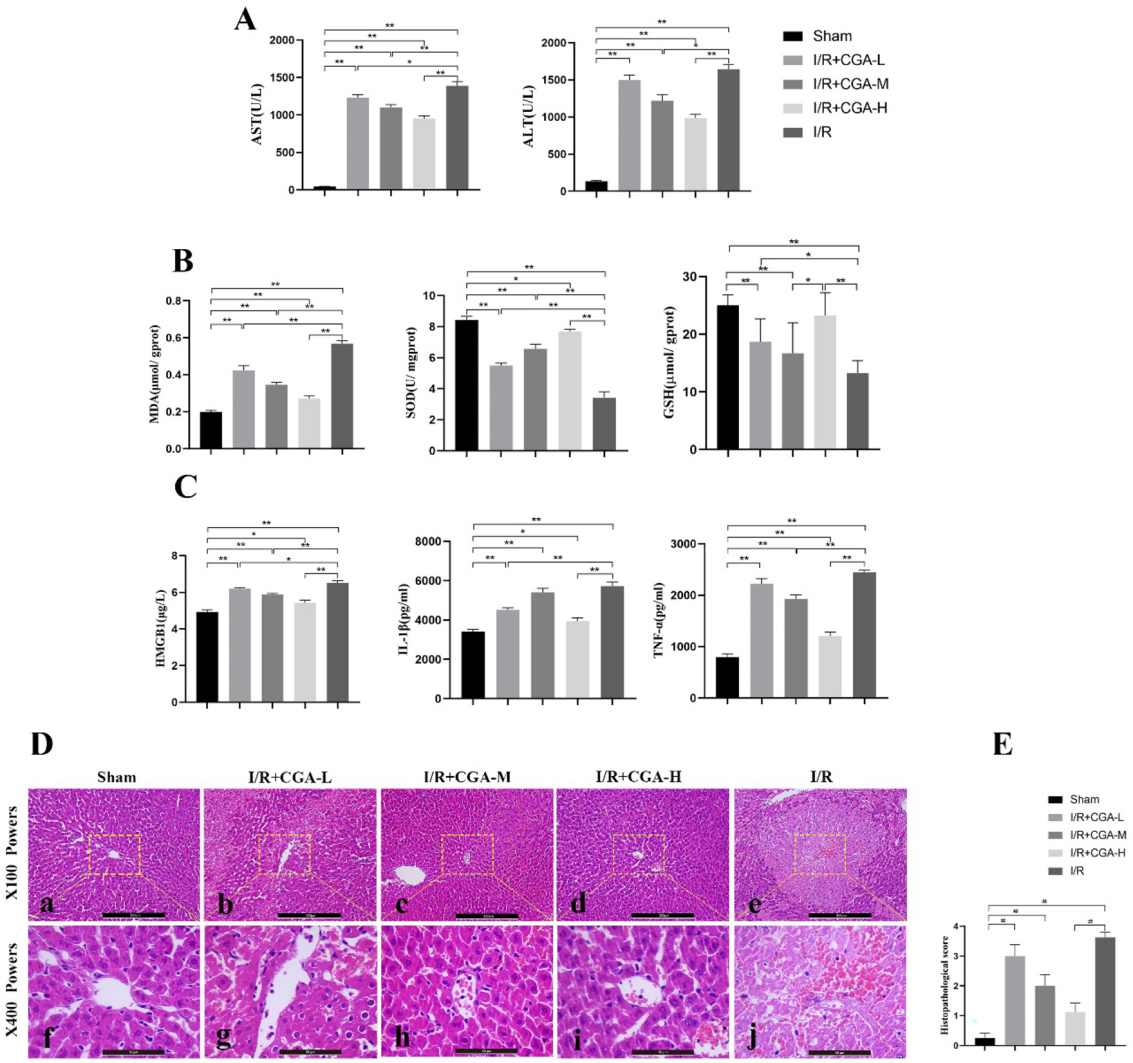
CGA pretreatment ameliorated liver function, oxidative stress, inflammation levels and the degree of liver histopathological damage in rats.

To demonstrate whether different concentrations of CGA pretreatment had a protective effect on HIRI and to obtain the optimal CGA concentration, we examined the levels of liver function, oxidative stress markers, inflammation factors, and the degree of liver histopathological damage in rats (Fig. 1). First, we assessed the effect of CGA on liver function by measuring its serum biomarker levels (Fig. 1A). The serum levels of ALT and AST were significantly increased after I/R injury compared with that of the Sham group (*p* < 0.01). In addition, compared with that of the I/R group, the serum levels of AST were significantly decreased after pretreatment with all doses of CGA; however, only medium and high doses of CGA pretreatment could decrease the ALT levels (*p* < 0.05). Therefore, CGA pretreatment could reverse liver function injury after HIRI and the most significant decrease was observed in the I/R+CGA-H group (*p* < 0.05).

To examine whether CGA affected oxidative stress in the liver, we measured liver homogenate levels of MDA, SOD and GSH (Fig. 1B). After HIRI, MDA levels increased, and SOD, GSH levels decreased significantly compared with that of the Sham group (*p* < 0.05). However, after CGA pretreatment, MDA levels decreased and SOD, GSH levels significantly increased compared to that of the I/R group (*p* < 0.05). This indicates that CGA pretreatment could reduce oxidative stress in the liver and this effect was most significant in the I/R + CGA-H group (*p* < 0.01).

Next, we investigated the effect of CGA on liver inflammation by measuring the liver homogenate levels of HMGB1, TNF-α, and IL-1β (Fig. 1C). The levels of HMGB1, TNF-α, and IL-1β were significantly increased after HIRI compared to that of the Sham group (*p* < 0.05). In particular, compared to that of the I/R group, HMGB1 levels were significantly decreased after all doses of CGA pretreatment (*p* < 0.05); however, only low and high doses of CGA pretreatment resulted in a significant decrease in IL-1β levels (*p* < 0.01), while only the medium and high doses of CGA pretreatment resulted in a significant decrease in TNF-α levels (*p* < 0.01). Therefore, CGA pretreatment could significantly decrease aseptic inflammation caused by HIRI and the most significant decrease was observed in the I/R + CGA-H group (*p* < 0.01).

Later, to investigate the histopathological effect of CGA on the liver, we performed H&E staining on liver tissues (Fig. 1D). The results revealed that hepatocytes had a normal pathological structure in the Sham group (Fig. 1D: a, f); however, those in the I/R group displayed hepatocyte edema, disorganized hepatic cord structure, vacuolation, lamellar necrosis, hemorrhage, and inflammatory cell infiltration (Fig. 1D: e, j). In contrast, hepatocellular lesions were reduced in the CGA groups (Fig. 1D: b–d, g–i). Kruskal–Wallis analyses of the histopathological scores between the different groups revealed that the low and medium CGA doses and I/R groups scored higher than the Sham group and that only the I/R + CGA-H group had a lower score compared to that of the I/R group (*p* < 0.005; Fig. 1E).

Since the highest CGA dose (100 mg/kg.d) exerted the strongest effect, it was used for subsequent experiments.

### CGA Reduced Liver ROS Levels in Rats

**Fig. 2:**
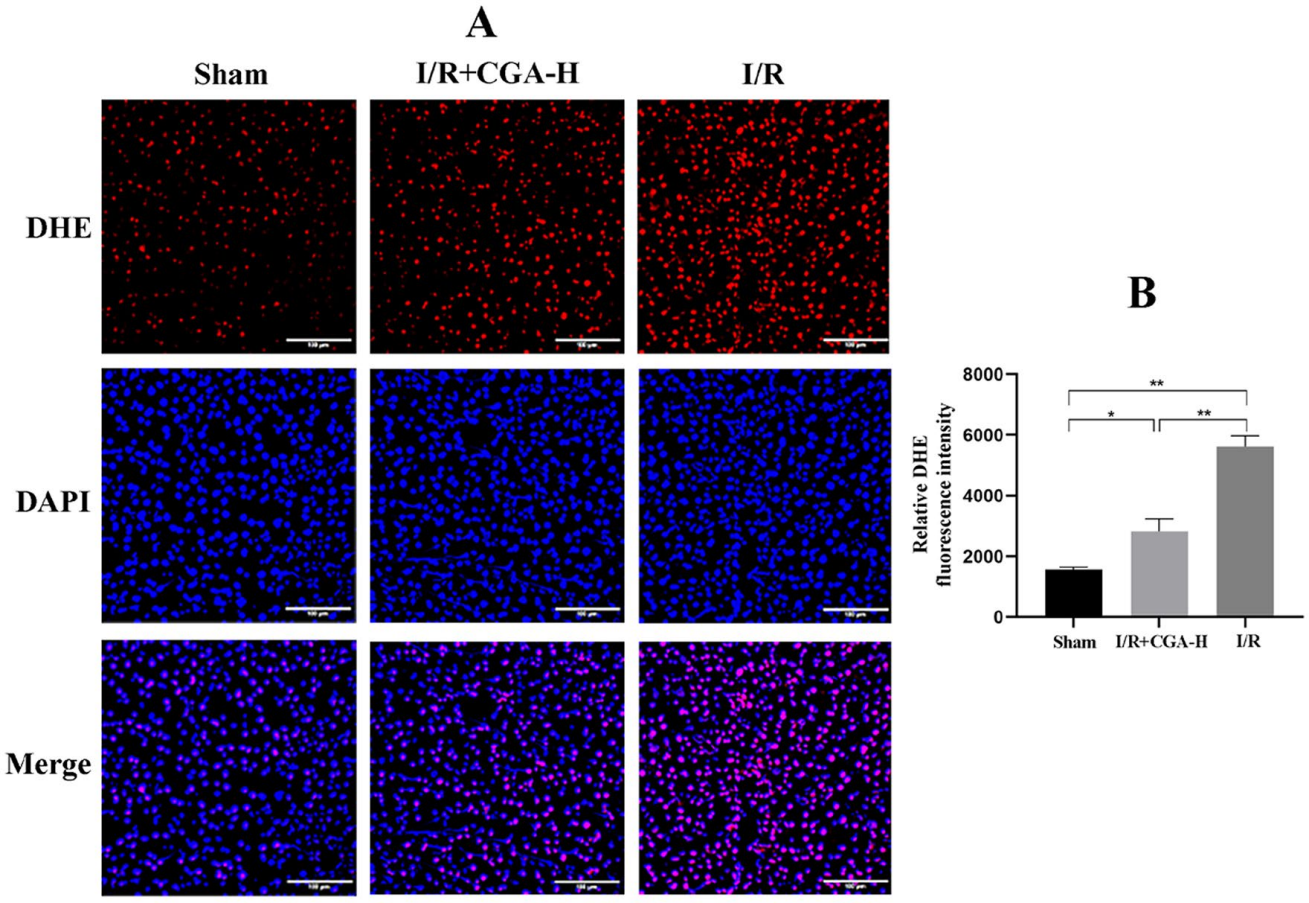
CGA pretreatment reduced ROS levels in rats.

To examine the effect of CGA on cellular ROS levels in rat liver tissue, we stained frozen liver sections from each group with a DHE fluorescent probe and DAPI (Fig. 2A, B). Interestingly, compared to that of the Sham group, DHE fluorescence intensity significantly increased in rats subjected to HIRI (*p* < 0.05) and decreased after CGA pretreatment, compared to that in the I/R group (*p* < 0.01). The results show that CGA could inhibit ROS production during HIRI in rats, which may in turn reduce oxidative stress-induced liver injury.

### CGA Reduced the Active Secretion of HMGB1 *In Vivo*

**Fig. 3:**
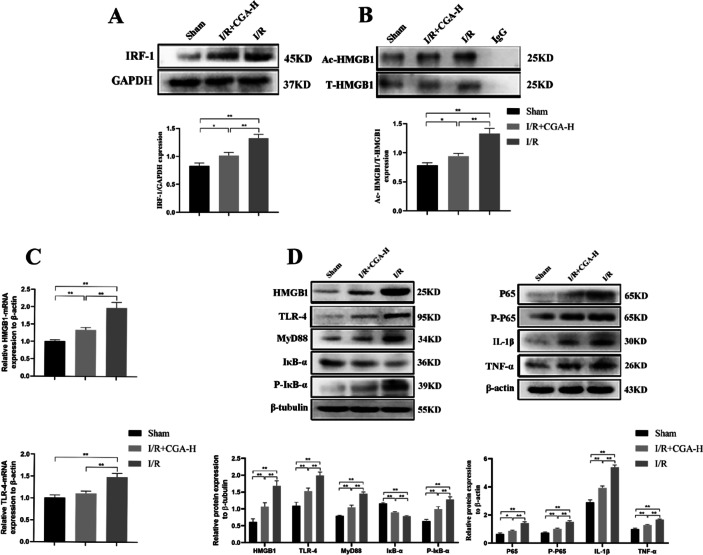
CGA attenuates the expression levels of inflammatory pathway related proteins in rats.

The expression of IRF-1 protein in the liver tissue of rats in each group was detected by western blotting, and Ac-HMGB1 expression in the liver tissue of rats in each group was detected by IP combined with western blotting. The results showed that IRF-1 and Ac-HMGB1 expression levels in the liver tissue of the I/R+CGA and I/R groups were increased compared with that of the Sham group (*p* < 0.05). However, the expression levels of IRF-1 and Ac-HMGB1 in the I/R+CGA group were significantly decreased compared with that of the I/R group (*p* < 0.01, Fig. 3A, B). The results suggest that CGA can effectively inhibit the active secretion of HMGB1 during HIRI in rats.

### CGA Decreased the Levels of HMGB1/TLR-4/NF-κB Signaling Pathway Components *In Vivo*

RT-qPCR analysis revealed that the mRNA levels of *HMGB1* and *TLR-4* in the I/R+CGA group were significantly lower than those in the I/R group (*p* < 0.01), among which the *TLR-4* mRNA levels were not significantly different from that in the Sham group (*p* > 0.05, Fig. 3C). The results suggest that CGA could inhibit the expression of HMGB1 and its downstream inflammatory pathway receptor, TLR-4, at the transcriptional level during HIRI in rats.

Next, to further determine the mechanism underlying the observed effects of CGA *in vivo*, we examined the expression of the TLR-4/NF-κB signaling pathway components in the liver tissues of each group (Fig. 3D). Compared with that of the Sham group, the protein expression of TLR-4, MyD88, P65, P-P65, P-IκB-α, IL-1β, and TNF-α was significantly increased after HIRI (*p* < 0.05), whereas the protein expression of IκB-α decreased. Moreover, the expression of TLR-4, MyD88, P65, P-P65, P-IκB-α, IL-1β, and TNF-α was significantly decreased after CGA pretreatment (*p* < 0.01) and the expression of IκB-α was significantly increased compared to that of the I/R group (*p* < 0.01). The results show that CGA could inhibit the NF-κB signaling pathway mediated by TLR-4, and thus reduce the sterile inflammatory response during HIRI in rats.

### CGA Attenuated Apoptosis in Rat Liver Tissue

**Fig. 4:**
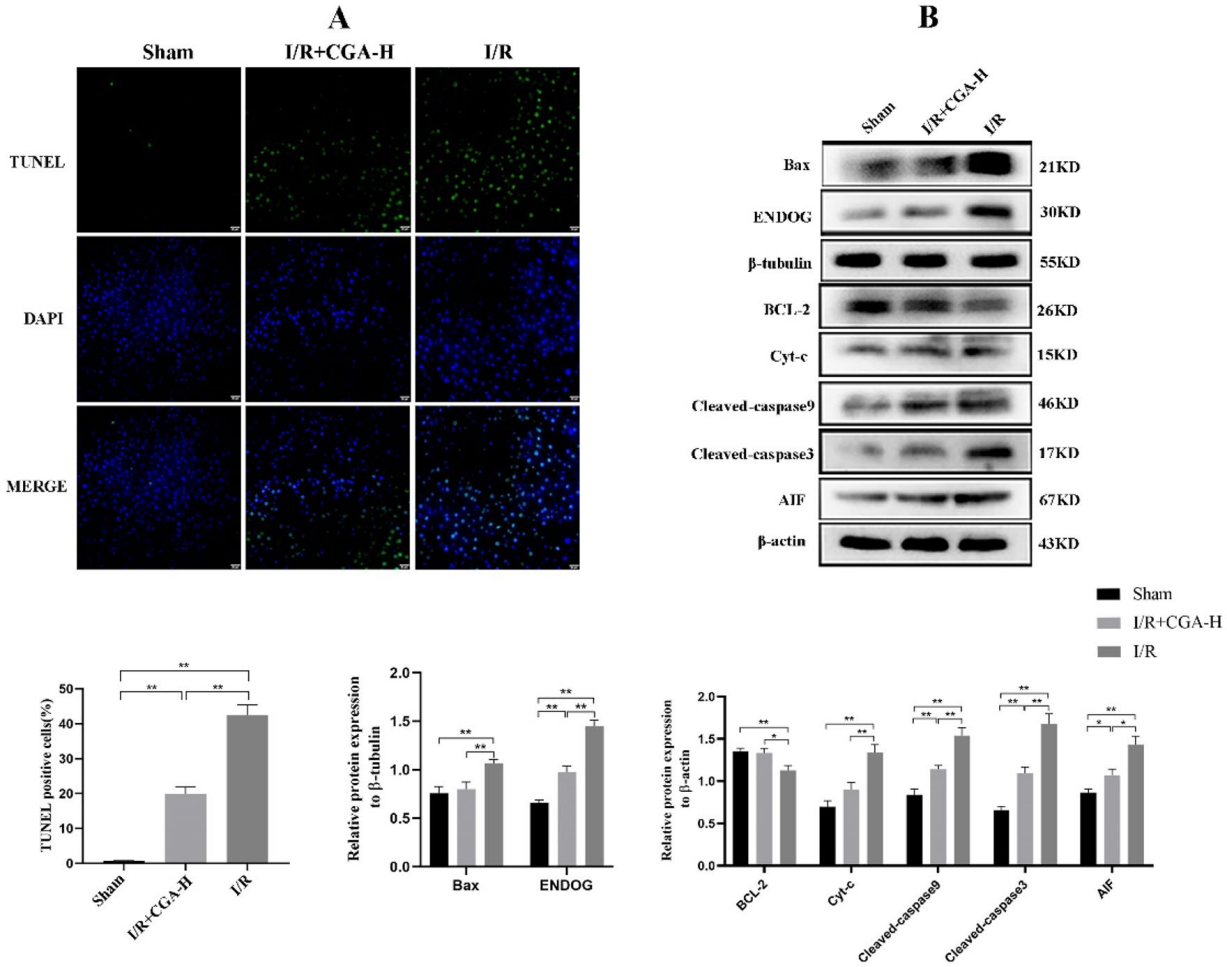
CGA pretreatment reduced hepatocyte apoptosis and the expression levels of mitochondria-mediated apoptosis pathway related proteins in rats.

First, we examined the effect of CGA on apoptosis by performing TUNEL staining on liver tissues (Fig. 4A). The results showed that apoptotic cells were significantly increased after I/R, compared to that of the Sham group, but were significantly reduced after CGA pretreatment (*p* < 0.01). We observed apoptotic cells after HIRI; however, it is unknown whether this is due to a mitochondria-mediated apoptotic pathway? Therefore, we further examined mitochondrial apoptosis pathway-related proteins to prove our hypothesis (Fig. 4B). Compared to that of the Sham group, BCL-2 expression significantly decreased after HIRI (*p* < 0.01), and its expression significantly increased after CGA pretreatment compared to that of the I/R group (*p* < 0.05). Conversely, Bax, Cyt-c, cleaved-caspase9, cleaved-caspase3, ENDOG, and AIF expression significantly increased after I/R injury compared to that of the Sham group (*p* < 0.01); however, their expression significantly decreased after CGA pretreatment compared to that of the I/R group (*p* < 0.05). The results indicate that CGA significantly attenuated the occurrence of hepatocyte apoptosis during HIRI in rats by inhibiting both the mitochondria-mediated caspase-dependent apoptotic and caspase-independent apoptotic pathways. It remains to be further explored in *in vitro* experiments whether the underlying cause is related to the reduction of hepatocyte mitochondrial damage by CGA.

### CGA Decreased the Levels of HMGB1/TLR-4/NF-κB Signaling Pathway Components *In Vitro*

**Fig. 5:**
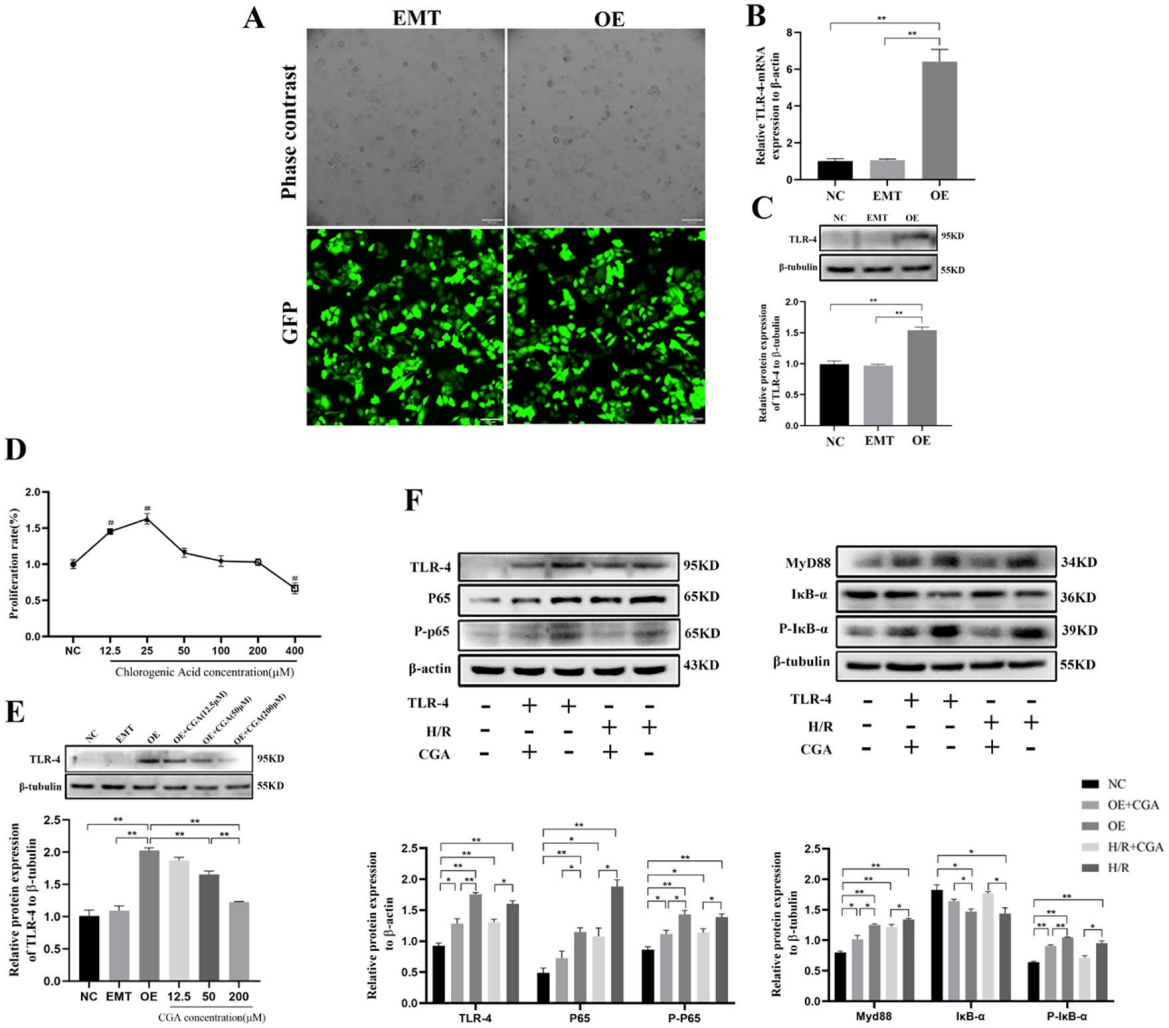
CGA decreased the levels of TLR-4/NF-κB signaling pathway components *in vitro*.

L02 cell lines overexpressing stable TLR-4 were screened using lentiviral transfection, and GFP expression was observed under a fluorescence microscope (Fig. 5A). RT-qPCR and western blotting were used to detect TLR-4 expression in the normal L02 cell (NC), empty plasmid transfection (EMT), and TLR-4 overexpression (OE) groups. The results showed that TLR-4 mRNA and protein levels were significantly upregulated in the OE group compared to the NC and EMT groups (*p* < 0.01), while the results in the EMT and NC groups were not significantly different (*P* > 0.05, Fig. 5B, C), indicating that the transfection was successful and the empty plasmid had no effect on the results of this experiment.

CCK8 results showed no toxic effect of 12.5–200 µM CGA on L02 cells, while 12.5–25 µM CGA promoted their proliferation (Fig. 5D). To determine the optimal CGA concentration, cells were divided into NC, EMT, OE, and OE + CGA (12.5, 50, or 200 µM) groups according to the CCK-8 assay results. The OE + CGA groups were administered 12.5, 50, and 200 µM CGA and cultured with TLR-4 overexpressing cells for 48 h. The protein expression of TLR-4 was detected by western blotting, revealing that the TLR-4 expression was significantly lower in the 50 and 200 µM CGA groups than in the OE group (*p* < 0.01). TLR-4 expression was particularly significant in the CGA 200 μM group (*p* < 0.01, Fig. 5E); therefore, CGA 200 µM was selected for subsequent experiments.

Next, we measured the protein expression of the TLR-4/NF-κB signaling pathway components in L02 cells (Fig. 5F). Compared to the protein levels in the NC group, TLR-4, MyD88, P65, P-P65 and P-IκB-α protein levels were significantly increased in the OE and H/R groups (*p* < 0.01), whereas the IκB-α expression was decreased (*p* < 0.05). Interestingly, these expression patterns were significantly reversed in the OE + CGA and H/R + CGA groups compared to that of the OE and H/R groups (*p* < 0.05). These results further indicate that CGA could reduce HIRI-induced sterile inflammation by inhibiting the TLR-4/NF-κB signaling pathway.

### CGA Reduced ROS Expression, Mitochondrial Damage, and Apoptosis *In Vitro*

**Fig. 6:**
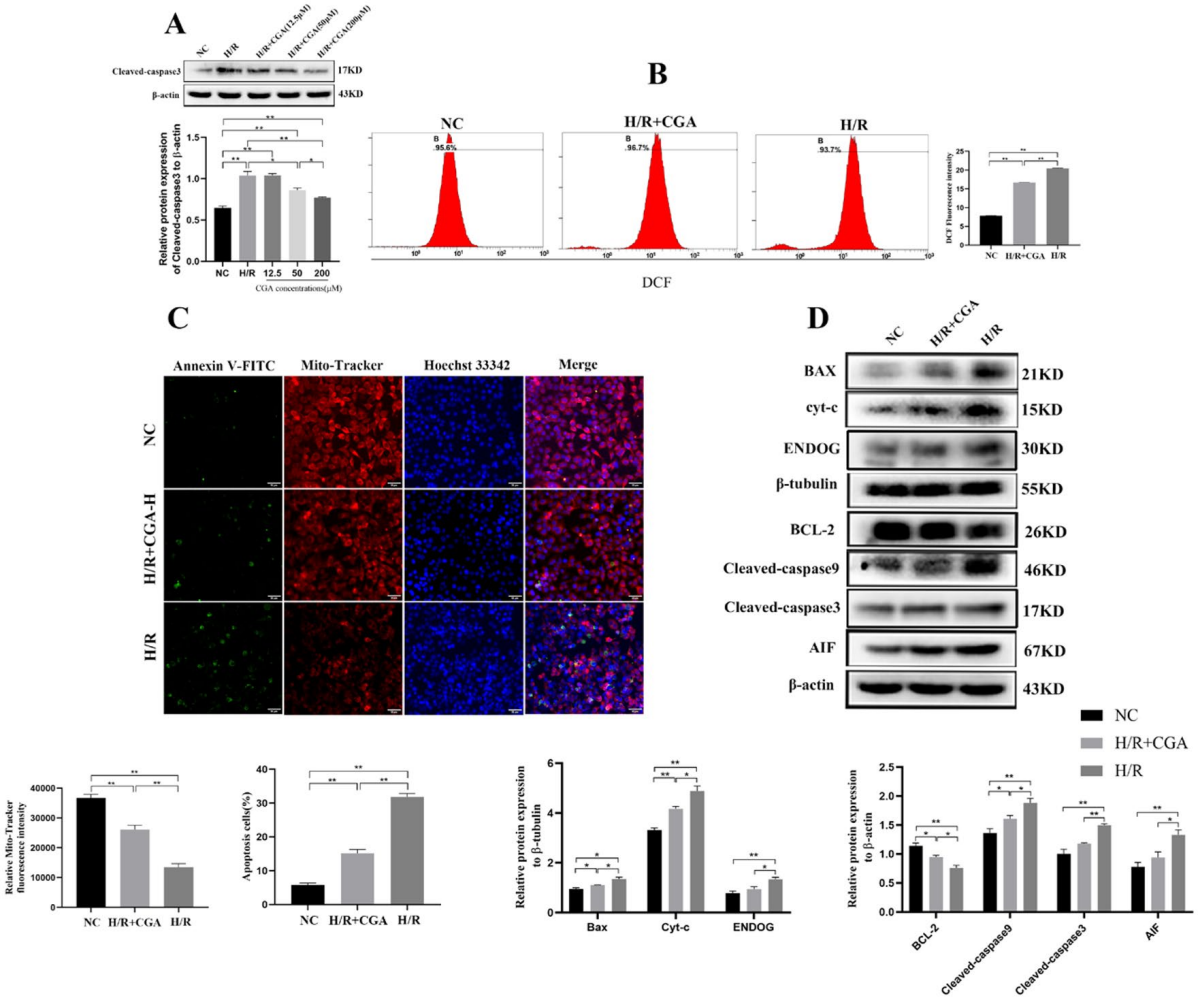
CGA reduced ROS expression, mitochondrial damage, and apoptosis *in vitro*.

To further prove our hypothesis, we established a hepatocyte hypoxia model for 6 h and reoxygenation model for 12 h to simulate HIRI, and examined ROS levels, mitochondrial damage, and apoptosis related indicators. First, L02 cells were divided into NC, H/R, and H/R+CGA low, medium, and high dose groups, the latter were pretreated with 12.5, 50 and 200 µM CGA for 48 h. The H/R and H/R+CGA groups were established as H/R models, and cleaved**-**caspase3 protein was detected in each group by western blotting. The results showed that the levels of cleaved**-**caspase3 protein in the H/R and H/R+CGA groups were significantly higher than those in the NC group (*p* < 0.05), and the levels of cleaved**-**caspase3 protein in the H/R+CGA 50 and 200 µM groups were significantly lower than those in the H/R group (*p* < 0.05), most significantly in the 200 µM group (*p* < 0.01, Fig. 6A). The results suggest that both medium and high doses of CGA may have protective effects on H/R-induced apoptosis, with the best effect observed at a CGA concentration of 200 µM. Therefore, 200 µM CGA was chosen for subsequent experiments to explore the specific underlying mechanism.

Next, L02 cells were stained with a DCFH-DA fluorescent probe and detected by flow cytometry (Fig. 6B). The ROS fluorescence intensity was higher in the H/R + CGA and H/R groups than in the NC group (*p* < 0.01). Furthermore, the ROS fluorescence intensity was decreased in the H/R + CGA group compared to that of the H/R group (*p* < 0.01). The results suggest that CGA inhibits the production of ROS in hepatocytes during H/R.

Next, MitoTracker Red CMXRos and Annexin V-FITC fluorescent probes were used to detect the mitochondrial damage and apoptosis of L02 cells (Fig. 6C). Compared to that of the NC group, MitoTracker fluorescence intensity in the H/R + CGA and H/R groups was significantly lower, whereas the apoptotic cells were significantly higher (*p* < 0.01). Conversely, MitoTracker fluorescence intensity was increased, and apoptosis was lower in the H/R + CGA group than in the H/R group (*p* < 0.01). We also detected the expression of mitochondrial apoptosis pathway-related proteins (Fig. 6D), and found that BCL-2 expression was significantly lower in the H/R group than in the NC group (*p* < 0.05). However, BCL-2 was significantly upregulated in the H/R + CGA group (*p* < 0.05) compared to that of the H/R group. Compared with that of the NC group, Bax, Cyt-c, cleaved-caspase9 and cleaved- caspase3, ENDOG, and AIF expression was significantly increased in the H/R group (*p* < 0.05); however, their expression was significantly decreased in the H/R + CGA group compared to that in the H/R group (*p* < 0.05). The results show that CGA could alleviate hepatocyte mitochondrial damage by reducing ROS production in hepatocytes during HIRI, thereby inhibiting the mitochondria-mediated apoptotic pathway and reducing hepatocyte injury.

## DISCUSSION

HIRI is an important cause of liver failure after liver surgery or transplantation; however, its underlying mechanism is highly complex. Kupffer cells are liver macrophages that play an important role in the development of HIRI. During the early stages of reperfusion after ischemia, Kupffer cells alter their metabolic behavior and produce a large amount of ROS [34]. In addition, endogenous antioxidants such as SOD and GSH are inactivated or removed during I/R, leading to reduced ROS scavenging [1]. Increased ROS levels can damage macromolecules and lipids and produce MDA. In addition, large amounts of ROS cause cellular chain reactions leading to inflammatory responses, cell death, and even organ failure [35]. Du Y et al. [36] showed that aloin and γ-oryzanol, which have antioxidant effects, had protective effects against HIRI, and the mechanism was related to ROS scavenging and increasing SOD and GSH levels. CGA also alleviated cerebral I/R injury in a rat model, and its mechanism is linked to an increase in SOD and GSH levels and a decrease in ROS and MDA levels [37]. In this study, we demonstrated that the damage following HIRI is caused by ROS. Notably, serum ALT and AST levels increased, SOD and GSH levels decreased, and ROS and MDA levels increased following HIRI. In addition, liver tissues displayed cellular edema, vacuolation, lamellar necrosis, massive inflammatory cell infiltration, and extensive hemorrhage associated with inflammation and apoptosis. Furthermore, CGA pretreatment decreased the levels of ALT, AST, MDA, and ROS while increasing SOD and GSH levels, as well as alleviating the pathological damage of liver tissue, suggesting that CGA has good antioxidant effects.

In our study, following HIRI, inflammatory markers such as HMGB1, TNF-α, and IL-1β were elevated, and hepatocytes were injured, showing that inflammatory processes were responsible for cellular injury. HMGB1 has been linked to the onset of sterile inflammation in numerous studies [38]. In a sepsis model, CGA also protects mice from sepsis by blocking the release of HMGB1 [39]. In this study, we found that HMGB1 expression was significantly increased in liver tissue after HIRI, and that TNF-α, IL-1β expression was also increased. These effects could be reversed by CGA, suggesting that CGA may have an anti-inflammatory effect in HIRI. As previously mentioned, HMGB1 normally binds to nuclear DNA in its deacetylated form; however, during I/R, it is acetylated (Ac-HMGB1) and secreted extracellularly, and ROS and interferon regulatory factor 1 (IRF-1) are involved in the secretion and translocation of HMGB1. Aucubin was reported to inhibit ROS and IRF-1 expression during HIRI in rats, resulting in reduced levels of HMGB1 and Ac-HMGB1 [23]. In this study, we found that ROS, IRF-1, and Ac-HMGB1 levels were significantly increased in the rat after HIRI, and their levels were significantly decreased following CGA pretreatment. This suggests that CGA can protect against HIRI by inhibiting the ROS/IRF-1 pathway and thus reducing the early active secretion of HMGB1.

Most studies have shown that HMGB1 promotes inflammation by binding to TLR-4 receptors and activating downstream pathways [40, 41]. Moreover, the TLR-4/NF-κB signaling pathway-mediated inflammation is vital in the development of HIRI [42]. In this process, activated TLR-4 receptor binds to the myeloid differentiation factor 88 (MyD88) adaptor protein, which in turn activates the nuclear factor kappa-B P65 (NF-κB P65, P65) signaling pathway and induces the expression of inflammatory factors such as IL-1β and TNF-α [43]. Furthermore, the blockade of TLR-4 expression may help alleviate HIRI. In this study, we showed that the protein levels of the TLR-4/NF-κB pathway (TLR-4, MyD88, P65, P-P65, P-IκB-α, TNF-α, and IL-1β) increased following HIRI in the rat model, whereas the IκB-α level decreased. However, CGA pretreatment was able to reverse these effects, indicating that CGA may act via the TLR-4/NF-κB pathway. To verify this result, we examined the effect of L02 cells overexpressing TLR-4 and L02 cells subjected to H/R to simulate HIRI. Both TLR-4 overexpression and H/R injury increased TLR-4, MyD88, P65, P-P65, and P-IκB-α levels and decreased IκB-α levels, whereas CGA pretreatment decreased TLR-4, MyD88, P65, P-P65, and P-IκB-α levels, and increased IκB-α levels, suggesting that CGA can protect against HIRI by alleviating the inflammatory response via the HMGB1/TLR-4/NF-κB axis.

Recent evidence has indicated that apoptosis is the main mode of I/R-induced hepatocyte death [14, 44]. ROS-induced oxidative stress plays a key role in cell apoptosis and promotes mitochondrial damage. In this research, we discovered a large rise in ROS following HIRI, as well as a corresponding increase in apoptotic cells in both *in vivo* and *in vitro* experiments, and a significant decrease after pretreatment with CGA, suggesting that CGA alleviated hepatocyte apoptosis. Recent studies have revealed that mitochondrial function plays a significant role in HIRI development [15, 45]. In addition, animal studies have shown that infusing active mitochondria before reperfusion can dramatically reduce HIRI and that blocking apoptosis can minimize the degree of I/R injury in the liver and heart [16, 17, 46]. In our research, we found that after H/R, mitochondria were greatly reduced and apoptotic cells increased, which was reversed by CGA pretreatment, implying that CGA may protect against HIRI by reducing mitochondrial damage. Furthermore, the BCL-2 protein family regulates the intrinsic apoptosis pathway, with certain members being pro-apoptotic (Bad and Bax) and others being anti-apoptotic (BCL-2 and BCL-XL). The susceptibility of cells to apoptotic stimuli is determined by the balance of these proteins [20]. Chitosan pretreatment was found to protect rats from HIRI by increasing BCL-2 expression while decreasing Bax expression [47]. In addition, Bax knockout mice livers have better tolerance to I/R injury [48]. In this study, we found that after HIRI, BCL-2 expression decreased and ROS and Bax increased *in vivo* and *in vitro*, whereas CGA pretreatment reversed this effect. This suggests that CGA protects mitochondria by reducing ROS production as well as affecting BCL-2 and Bax expression.

Mitochondrial damage occurs when mitochondria are continuously stimulated by ROS and pro-inflammatory factors, thereby disrupting the balance between proapoptotic and antiapoptotic factors. Pro-apoptotic proteins initiate apoptosis by forming mitochondrial outer membrane permeability complexes that release Cytochrome C (Cyt-c) into the cytoplasm [49]. This then activates the caspase cascade to generate apoptosis bodies and initiate endogenous apoptosis. Several studies have recently shown that inhibiting Cyt-c spillover and caspase activation could help alleviate HIRI [50, 51]. In our study, we found that HIRI boosted Cyt-c overflow and activated downstream caspases (caspase9, cleaved-caspase9, caspase3, cleaved-caspase3). CGA pretreatment decreased Cyt-c spillover and reduced caspase activation, thereby alleviating hepatocyte apoptosis caused by the mitochondria-mediated caspase-dependent pathway *in vivo* and *in vitro*. Mitochondria also mediate the caspase-independent apoptosis pathway and play an important role in the mitochondrial apoptosis pathway, including ENDOG and AIF, which can cause chromatin DNA cleavage, chromatin condensation, and DNA degradation [52, 53]. Recently, in a model of cerebral I/R injury, the Gualou Guizhi decoction, an herbal medicine, could protect against brain injury by inhibiting the expression of ENDOG and AIF. In our study, we found that ENDOG and AIF were upregulated after HIRI but downregulated after CGA pretreatment *in vivo* and *in vitro*, thereby alleviating hepatocyte apoptosis.

Together, we have proved that oxidative stress, aseptic inflammation and mitochondria mediated apoptosis are the causes of HIRI and the protective effect of CGA on HIRI *in vivo* and *in vitro*. However, there are still shortcomings in our experiments, such as not detecting the levels of IRF-1 and AC-HMGB1 at the cellular level, and not upregulating the mitochondrial apoptosis pathway by the over-expression of agonists or key proteins. These observations provide a comprehensive overview of the protective mechanism of CGA against HIRI and suggest that CGA could be used to treat HIRI; however, further studies are warranted to elucidate the detailed underlying mechanism.

## CONCLUSION

**Fig. 7:**
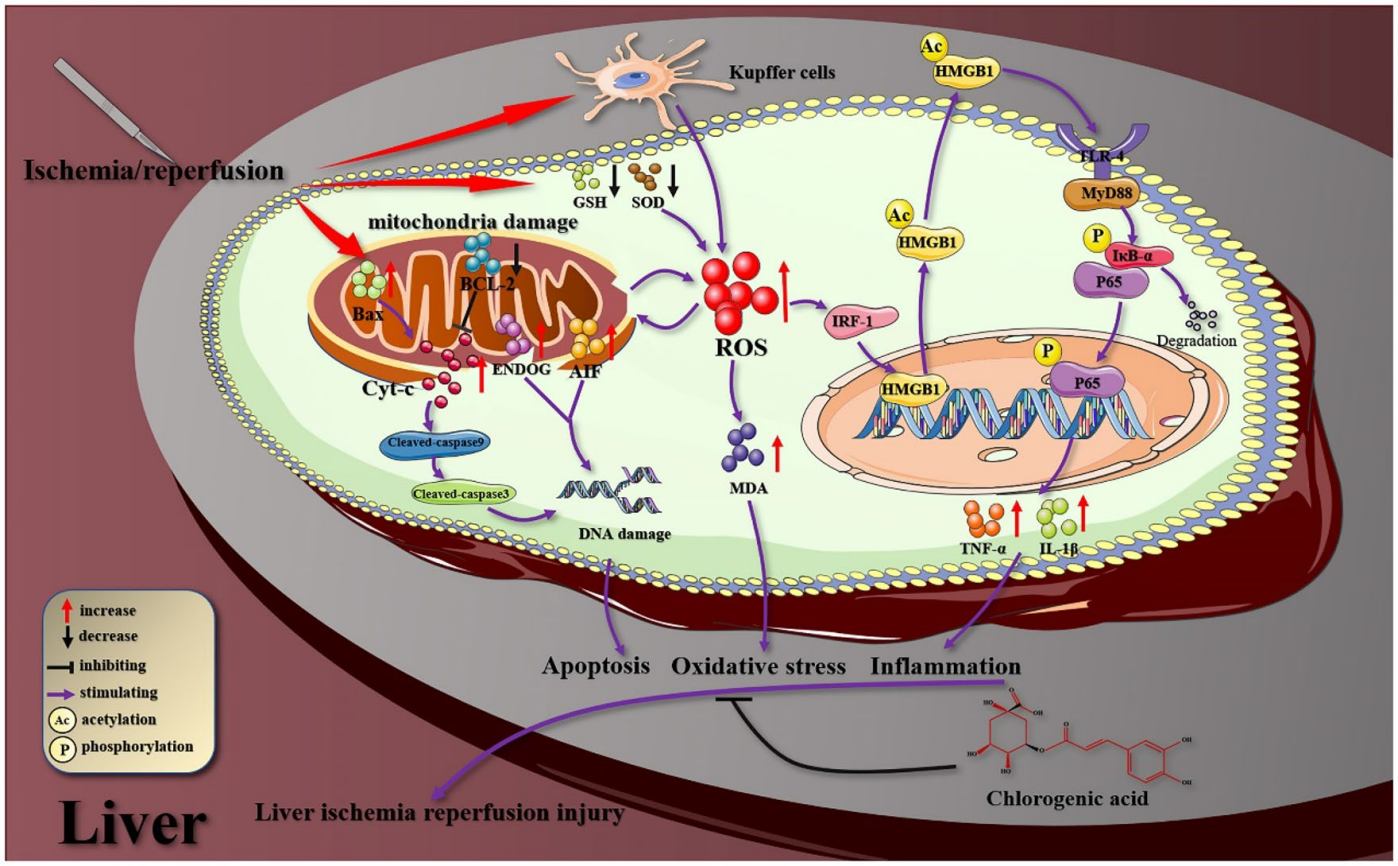
A potential mechanism model for CGA regulation of oxidative stress, inflammatory response and mitochondria-mediated apoptosis in HIRI.

In conclusion, the findings of this study demonstrate that CGA can decrease HIRI (Fig. 7) by (1) reducing liver transaminase activity and histopathological changes in the liver; (2) inhibiting oxidative stress; (3) reducing the active secretion of HMGB1 and regulating TLR-4/NF-κB signaling pathway to reduce the expression of inflammatory factors; and (4) inhibiting the mitochondria-mediated apoptosis pathway.

## Supplementary Information

Below is the link to the electronic supplementary material.

Supplementary file1 (DOCX 106 KB)

## Data Availability

The experimental data used to support this study can be provided upon request.

## Abbreviations

AIF: Apoptosis inducing factor
ALT: Alanine aminotransferase
AST: Aspartate aminotransferase
Bax: BCL2-Associated X
BCL-2: B-cell lymphoma-2
CGA: Chlorogenic acid
Cyt-c: Cytochrome C
ENDOG: Endonuclease G
GSH: Glutathione
HIRI: Hepatic ischemia–reperfusion injury
HMGB1: High mobility group protein B1
IL-1β: Interleukin-1β
IRF-1: Interferon regulatory factors 1
IκB-α: I kappa B alpha
MDA: Malondialdehyde
MyD88: Myeloid differentiation factor 88
NF-κB P65: Nuclear factor kappa-B P65
P-IκB-α: Phospho-I kappa B alpha
P-P65: Phospho nuclear factor kappa-B P65
ROS: Reactive oxygen species
SOD: Superoxide dismutase
TLR-4: Toll-like receptors 4
TNF-α: Tumor necrosis factor alpha
