# Supplementary material for: Chlorogenic Acid Alleviates Hepatic Ischemia–Reperfusion Injury by Inhibiting Oxidative Stress, Inflammation, and Mitochondria-Mediated Apoptosis In Vivo and In Vitro
Source: Inflammation. 2023 Mar 1;46(3):1061–76. doi: 10.1007/s10753-023-01792-8 (PMC10188389; doi:10.1007/s10753-023-01792-8)
Supplement: Supplementary file 1 — Supplementary file1 (DOCX 106 KB) [file 10753_2023_1792_MOESM1_ESM.docx]

# SUPPLEMENTARY MATERIAL

**Fig S1:** Chemical structural formula of chlorogenic acid.


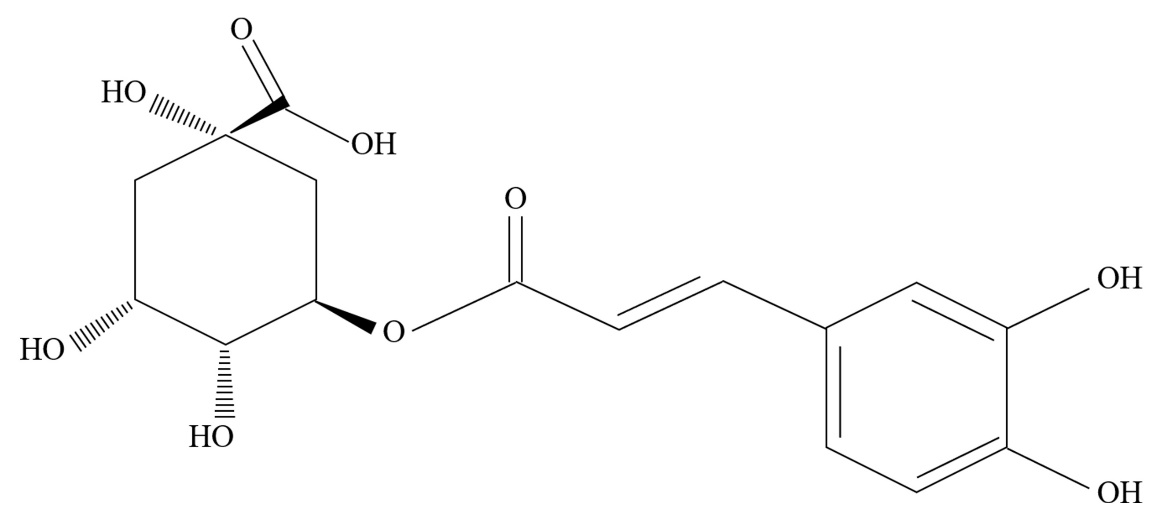


**Fig S2:** Rat liver I/R model.


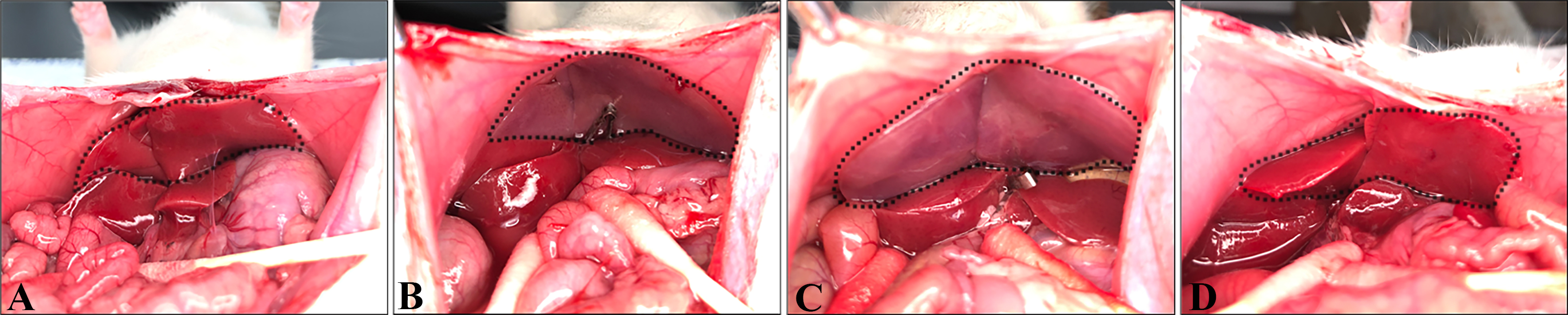


Rat liver I/R model (black dotted line represents part of liver with I/R). (A) The liver is not ischemic; (B) liver ischemia; (C) 1 hour after liver ischemia; (D) 4 hours after liver reperfusion.

**Tab S1:** Primer sequences of target genes *HMGB1*, *TLR-4* and β-actin.

| Target genes | Forward (5′-3′) | Reverse primer (3′-5′) |
| --- | --- | --- |
| *HMGB1* | 5′-ACAACACTGCTGCGGATGACAAG-3′ | 3′-CCTTCTCCTTCTGCTGCTCCTCC-5′ |
| *TLR-4* | 5′-GCTGCCAACATCATCCAGGAAGG-3′ | 3′-GACTCATCGGCGAGACCGTAGT-5′ |
| β-actin | 5′-CACTATCGGCAATGAGCGGTTCC-3′ | 3′-CTGGAGATACGGTTGTGTCACGAC-5′ |

**Tab S2:** Primary antibodie dilution ratio, suppliers, prefecture and countries.

| Protein | Bilution ratio | Supplier | Prefecture | Country |
| --- | --- | --- | --- | --- |
| TLR-4 | 1:1000 | Proteintech | Wuhan | CHINA |
| HMGB1 | 1:1000 | Proteintech | Wuhan | CHINA |
| MyD88 | 1:1000 | Proteintech | Wuhan | CHINA |
| IRF-1 | 1:1000 | Proteintech | Wuhan | CHINA |
| P65 | 1:1000 | Proteintech | Wuhan | CHINA |
| P-P65 | 1:1000 | Affinity Biosciences | Cincinnati | USA |
| IκB-α | 1:1000 | Proteintech | Wuhan | CHINA |
| P-IκB-α | 1:1000 | Affinity Biosciences | Cincinnati | USA |
| TNF-α | 1:1000 | Bioss Antibodies | Beijing | CHINA |
| IL-β | 1:1000 | Bioss Antibodies | Beijing | CHINA |
| BCL-2 | 1:1000 | Proteintech | Wuhan | CHINA |
| Bax | 1:1000 | Proteintech | Wuhan | CHINA |
| caspase9 | 1:1000 | Proteintech | Wuhan | CHINA |
| cleaved-caspase9 | 1:1000 | Proteintech | Wuhan | CHINA |
| cytochrome C | 1:1000 | Proteintech | Wuhan | CHINA |
| caspase3 | 1:1000 | Proteintech | Wuhan | CHINA |
| cleaved-caspase3 | 1:1000 | Proteintech | Wuhan | CHINA |
| ENDOG | 1:1000 | Proteintech | Wuhan | CHINA |
| AIF | 1:1000 | Proteintech | Wuhan | CHINA |
| β-actin | 1:4000 | Proteintech | Wuhan | CHINA |
| β-tubulin | 1:4000 | Proteintech | Wuhan | CHINA |
| GAPDH | 1:4000 | Proteintech | Wuhan | CHINA |
